# Serum IL-17 levels are higher in critically ill patients with AKI and associated with worse outcomes

**DOI:** 10.1186/s13054-022-03976-4

**Published:** 2022-04-14

**Authors:** Jason A. Collett, Victor Ortiz-Soriano, Xilong Li, Alexander H. Flannery, Robert D. Toto, Orson W. Moe, David P. Basile, Javier A. Neyra

**Affiliations:** 1 Department of Anatomy, Cell Biology and Physiology, Indiana University School of Medicine, Indianapolis, IN USA; 2 Division of Nephrology, Department of Internal Medicine, Bone and Mineral Metabolism, University of Kentucky Medical Center, University of Kentucky, 800 Rose St., MN668, Lexington, KY 40536 USA; 3 Charles and Jane Pak Center for Mineral Metabolism and Clinical Research, University of Texas Southwestern Medical Center, Dallas, TX USA; 4 Department of Pharmacy Practice and Science, University of Kentucky College of Pharmacy, Lexington, KY USA; 5 Division of Nephrology, Department of Internal Medicine, University of Texas Southwestern Medical Center, Dallas, TX USA

**Keywords:** IL-17, Acute kidney injury, Critical care, Mortality, Major adverse kidney events

## Abstract

**Background:**

Interleukin-17 (IL-17) antagonism in rats reduces the severity and progression of AKI. IL-17-producing circulating T helper-17 (TH17) cells is increased in critically ill patients with AKI indicating that this pathway is also activated in humans. We aim to compare serum IL-17A levels in critically ill patients with versus without AKI and to examine their relationship with mortality and major adverse kidney events (MAKE).

**Methods:**

Multicenter, prospective study of ICU patients with AKI stage 2 or 3 and without AKI. Samples were collected at 24–48 h after AKI diagnosis or ICU admission (in those without AKI) [timepoint 1, T1] and 5–7 days later [timepoint 2, T2]. MAKE was defined as the composite of death, dependence on kidney replacement therapy or a reduction in eGFR of ≥ 30% from baseline up to 90 days following hospital discharge.

**Results:**

A total of 299 patients were evaluated. Patients in the highest IL-17A tertile (versus lower tertiles) at T1 had higher acuity of illness and comorbidity scores. Patients with AKI had higher levels of IL-17A than those without AKI: T1 1918.6 fg/ml (692.0–5860.9) versus 623.1 fg/ml (331.7–1503.4), *p* < 0.001; T2 2167.7 fg/ml (839.9–4618.9) versus 1193.5 fg/ml (523.8–2198.7), *p* = 0.006. Every onefold higher serum IL-17A at T1 was independently associated with increased risk of hospital mortality (aOR 1.35, 95% CI: 1.06–1.73) and MAKE (aOR 1.26, 95% CI: 1.02–1.55). The highest tertile of IL-17A (vs. the lowest tertile) was also independently associated with higher risk of MAKE (aOR 3.03, 95% CI: 1.34–6.87). There was no effect modification of these associations by AKI status. IL-17A levels remained significantly elevated at T2 in patients that died or developed MAKE.

**Conclusions:**

Serum IL-17A levels measured by the time of AKI diagnosis or ICU admission were differentially elevated in critically ill patients with AKI when compared to those without AKI and were independently associated with hospital mortality and MAKE.

**Supplementary Information:**

The online version contains supplementary material available at 10.1186/s13054-022-03976-4.

## Introduction

Acute kidney injury (AKI) frequently occurs in critically ill patients admitted to the intensive care unit (ICU) and is associated with high morbidity and mortality [1–7]. Although many ICU survivors recover from AKI, it is estimated that up to 25% develop acute kidney disease within the first 90 days of onset and one-third of survivors develop incident or progressive chronic kidney disease (CKD) within the next 5 years [8]. AKI typically occurs early in the course of ICU stay limiting primary prevention; therefore, it is crucial to develop risk-classification tools to assist with timely interventions that could mitigate AKI progression, promote kidney recovery, and improve survival.

Biomarker development, testing and validation have extensively focused on prediction and early detection of AKI. In contrast, evaluation of biomarker utility for the prediction of progression of AKI and other relevant clinical outcomes such as mortality or dependence on kidney replacement therapy (KRT) is limited [9–11]. It is well recognized that AKI associates with intrarenal and systemic inflammation [12]. In this context, the proinflammatory cytokine interleukin 17 (IL-17) has gained recognition due to its candidacy not only as a potential biomarker of AKI but also for its ability to sub-phenotype pathways of inflammation during AKI, which could constitute potential therapeutic targets for kidney repair [13].

IL-17A, commonly referred to as IL-17 is one of the six members of the IL-17 cytokine family that plays an important role in the pathogenesis of a variety of different kidney-related diseases, including, but not limited to transplant rejection, diabetic nephropathy, autoimmune diseases, hypertension, and CKD [13, 14]. CD4+ cells expressing the proinflammatory cytokine IL-17A (TH17 cells) are rapidly expanded following kidney injury. Interventions that mitigate AKI severity in rats manifest reduced kidney TH17 cell expression [15–18], while vitamin D deficiency in rats results in greater TH17 cell activity and exacerbation of AKI [19]. Moreover, we and others have demonstrated that IL-17 antagonism reduces the severity of AKI and the subsequent development of CKD in murine models [13, 20–22]. Thus, IL-17A may play a critical role in the pathophysiology of AKI.

Recently, we measured an increase in TH17 cells from circulating blood of critically ill patients with AKI versus without AKI indicating that this pathway is also activated in human AKI [13]. Based on these observations, the current study sought to address the hypothesis that serum levels of IL-17A are higher in critically ill adult patients with AKI than in those without AKI, and that higher IL-17A levels associate with increased risk of mortality and major adverse kidney events (MAKE).

## Methods

### Study design and participants

We conducted a multicenter, prospective study of critically ill patients admitted to the ICU at two large academic medical centers: the University of Texas Southwestern (UTSW) and the University of Kentucky (UKY) [23, 24]. Inclusion criteria consisted of adult ICU patients (≥ 18 years old) with a known baseline eGFR ≥ 60 ml/min/1.73 m^2^. The study enrolled ICU patients with AKI stages 2 or 3, defined by KDIGO [25] serum creatinine and urine output criteria and ICU patients without AKI that were frequency-matched to those with AKI by pre-specified criteria that included age (10-years intervals), gender (male or female), and baseline eGFR (≥ 90 and 60–89 ml/min/1.73 m^2^). If a patient was recruited as a control without AKI and later developed AKI, they were excluded from the study. The enrollment was done in three sequential batches of 50 patients with AKI and 50 frequency-matched patients without AKI. Patients were enrolled between November 2014 and September 2019. Exclusion criteria consisted of evidence of AKI prior to ICU admission (e.g., AKI diagnosed at the referring hospital or on the floor before transfer to the ICU), end-stage kidney disease (ESKD), uroepithelial tumors, or prior solid organ transplant.

Baseline serum creatinine (SCr) was defined as the most recent outpatient SCr within the 1-year period before ICU admission. Estimates of GFR were calculated by using the CKD-Epidemiology Collaboration (CKD-EPI) equation. The study was approved by the institutional review boards at both participating centers (UTSW: STU 112014-065 and UK: 16-0936-F1V). Patients, or their legally authorized representatives, provided written informed consent for participation in the study.

### Biospecimen collection

We interrogated two timepoints of biospecimen collection in the present study. The first sample (timepoint 1 or T1) was obtained at the time of enrollment, which was 24–48 h after AKI diagnosis (meeting criteria of KDIGO stage ≥ 2) or ICU admission for those without AKI. The second sample (timepoint 2 or T2) was obtained 5–7 days after the first collection. We restricted AKI cases to those occurring in the first 7 days of ICU stay. Standardized techniques for blood collection, transport, processing, and storage were employed. Blood biospecimens were centrifuged at 1000 g, 4 °C for 10 min. Serum supernatant was aliquoted in codified non-siliconized cryovials and stored at − 80 °C locally at each institution until biomarker measurements were done by laboratory personnel of the Biomarker Analysis Lab at the University of Kentucky Center for Clinical and Translational Science. All laboratory personnel were blinded to the study design and data.

### Laboratory analyses

Serum IL-17A measurements were obtained using a high sensitivity ELISA assay (S-PLEX^®^ Human IL-17A Kit; Meso Scale Discovery). The expected lower and upper limits of detection in serum are 13.36 and 235,000 fg/ml (coefficient of variation < 25%), respectively. All other laboratory data were extracted from routine measurements performed for clinical care that were available in the electronic health records (EHR).

### Clinical data

Demographic, comorbidity data, and ICU-centric data (cumulative fluid balance, need for mechanical ventilation, KRT, and vasopressor/inotrope support) were collected from the EHR. Patients’ comorbidities and severity of illness were further assessed with the Charlson Comorbidity Index, the Acute Physiology and Chronic Health Evaluation (APACHE) II score, and the Sequential Organ Failure Assessment (SOFA) score. For both APACHE II and SOFA scores, the points related to serum creatinine were subtracted from the total score. Anemia was defined as the admission hematocrit less than 39% for men and less than 36% for women.

### Study outcomes

The main study outcomes were hospital mortality and major adverse kidney events at 90 days following hospital discharge (MAKE) and were determined based on EHR data. MAKE encompassed a composite of mortality, dependence on KRT or a reduction in eGFR of ≥ 30% from baseline by the last point of observation up to 90 days following hospital discharge. First, we determined mortality events. Second, we determined dependence on KRT in all survivors. Third, for patients that were alive and not dependent on KRT, we used the last SCr in the EHR up to 90 days post-discharge to determine an eGFR drop of ≥ 30% from baseline. Secondary outcomes included total days in the hospital, total days in the ICU, and total days on mechanical ventilation. In a sensitivity analysis, a ≥ 50% reduction in the eGFR threshold for MAKE rather than 30% reduction was evaluated.

### Statistical analysis

Categorical data were reported as percentages and continuous data as mean ± standard deviation or median (interquartile range). Comparisons across IL-17A tertiles for categorical variables were made using Fisher exact test. For continuous variables, analysis of variance was used for Gaussian and Kruskal–Wallis test for non-Gaussian distributed data. Two-group comparisons were done using the Chi-square test for categorical data and the *t* test or Mann–Whitney U test for continuous data as appropriate. Data distribution was assessed by the Shapiro–Wilk normality test and normal probability plots. IL-17A data were non-Gaussian distributed and were therefore natural log transformed.

For each main outcome (hospital mortality and MAKE), multivariable logistic regression models were constructed using IL-17A measurements from first sample (T1) as the independent variable both continuous and stratified by tertiles. Incremental multivariable models that adjusted for measured confounders were constructed. Model 1 adjusted for age, gender, race, Charlson Comorbidity index, and baseline eGFR; model 2 included all variables in model 1 plus the APACHE II score and the study site. Model 3 included all variables from model 2 plus the serum creatinine measurement at sample collection. Finally, a mixed model for repeated measures was used to examine the two-timepoint measurements of IL-17A (T1 and T2) and their association with hospital mortality and MAKE. For secondary continuous outcomes, generalized linear regression models were evaluated using IL-17A measurements from first sample (T1) as the independent variable in the whole study population and in the subgroup of hospital survivors. A two-sided *p* value ≤ 0.05 was considered statistically significant. We used SAS 9.4 (SAS Institute, Cary, NC) for all statistical analyses.

## Results

### Clinical characteristics

Data and serum samples from 299 patients were available for analysis. The mean (SD) age of study patients was 56.8 (15.2), 41.5% were women and 78.6% white. Baseline clinical characteristics according to tertiles of the first serum IL-17A measurement are shown in Table 1. Patient demographics and baseline kidney function were not significantly different according to IL-17A tertiles. Comorbidity data, including Charlson scores, were comparable across IL-17A tertiles with the exception of liver disease, which was more prevalent in patients in the highest IL-17A tertile versus lower tertiles. Similarly, patients in the highest tertile of IL-17A had higher prevalence of anemia than those in the lower tertile (Table 1). Critical illness parameters were notably different between the tertiles of IL-17A. Among these, cumulative fluid balance and serum creatinine levels were higher in patients in the highest IL-17A tertile when compared to the lower tertiles. Similarly, requirement of mechanical ventilation was more frequent in patients in the highest IL-17A tertile than in those in the lower tertiles. Non-renal APACHE II and SOFA scores were also higher in patients in the highest IL-17A tertile when compared to patients in the lower tertiles (Table 1).

**Table 1:** Patient characteristics according to serum IL-17A tertiles

Characteristic	Tertile 1	Tertile 2	Tertile 3	*p* value
Range, fg/ml	≤ 571.7	586.9–2295.6	≥ 2313.9	
No. of patients	99	100	100	
Demographics				
Age, years ± SD	58.1 ± 14.9	57.0 ± 15.4	55.3 ± 15.4	0.447
Women, *n* (%)	49 (49.5)	34 (34.0)	41 (41.0)	0.085
Race, *n* (%)				
White	77 (77.8)	76 (76.0)	82 (82.0)	0.794
Black	11 (11.1)	10 (10.0)	7 (7.0)	
Other	11 (11.1)	14 (14.0)	11 (11.0)	
Body mass index, kg/m^2^	30.9 [24.9–35.9]	27.8 [24.0–36.28]	27.1 [22.9–34.9]	0.327
Baseline kidney function				
Baseline eGFR, ml/min/1.73 m^2^, median [IQR]	88.4 [73.1–99.0]	88.6 [75.7–101.9]	90.9 [80.6–102.1]	0.409
Baseline SCr, mg/dl, median [IQR]	0.9 [0.7–1.0]	0.9 [0.8–1.0]	0.8 [0.7–1.0]	0.624
Comorbidity				
Diabetes, *n* (%)	28 (28.3)	22 (22.0)	27 (27.0)	0.563
Hypertension, *n* (%)	52 (52.5)	50 (50.0)	53 (53.0)	0.901
CHF, *n* (%)	32 (32.3)	23 (23.0)	18 (18.0)	0.094^a^
Liver disease, *n* (%)	14 (14.1)	11 (11.0)	27 (27.0)	0.007^a,b^
Anemia*, *n* (%)	43 (43.4)	54 (54.0)	62 (62.0)	0.025^a^
Cancer, *n* (%)	34 (34.3)	21 (21.0)	26 (26.0)	0.091
Charlson comorbidity index, median [IQR]	3.0 [2.0–5.0]	3.0 [1.0–5.0]	3.0 [2.0–5.0]	0.501
Critical illness parameters				
SCr at first timepoint (t1), mg/dl, median [IQR]	0.9 [0.7–1.7]	1.0 [0.7–2.6]	2.0 [1.0–3.0]	< 0.001^a,b^
Acute kidney injury, *n* (%)	33 (33.3)	47 (47.0)	73 (73.0)	< 0.001^a,b^
CFB 72 h, L, median [IQR]	1.7 [− 0.4 to 4.1]	1.7 [0.2–4.6]	3.9 [0.4–7.6]	0.020^a,b^
Pressor or inotrope, *n* (%)	53 (53.5)	44 (44.0)	63 (63.0)	0.027^b^
Mechanical ventilation, *n* (%)	44 (44.4)	48 (48.0)	63 (63.0)	0.021^a,b^
Packed RBC transfusion, *n* (%)	28 (28.3)	34 (34.0)	53 (53.0)	0.001^a,b^
Non-renal APACHE II score, median [IQR]	14.0 [10.0–19.0]	15.5 [10.8–22.0]	19.0 [14.0–23.3]	< 0.001^a,b^
Non-renal SOFA score, median [IQR]	5.00 [2.0–8.0]	5.0 [2.0–9.3]	7.00 [4.0–11.0]	0.006^a,b^

*APACHE II* acute physiologic assessment and chronic health evaluation II score excluding the renal component, *CFB* cumulative fluid balance, *CHF* congestive heart failure, *eGFR* estimated glomerular filtration rate, *ICU* intensive care unit, *IQR* interquartile range, *RBC* red blood cell, *SCr* serum creatinine, *SD* standard deviation, *SOFA* sequential organ failure assessment score excluding the renal component

*Anemia was defined as hematocrit at admission less than 39% in males or 36% in females. Anemia was defined as the admission hematocrit less than 39% for men and less than 36% for women

^a^Denotes *p* < 0.05 for comparisons between highest and lowest tertiles

^b^Denotes *p* < 0.05 for comparisons between highest and middle tertiles

### IL-17A levels and AKI

**Fig. 1:**
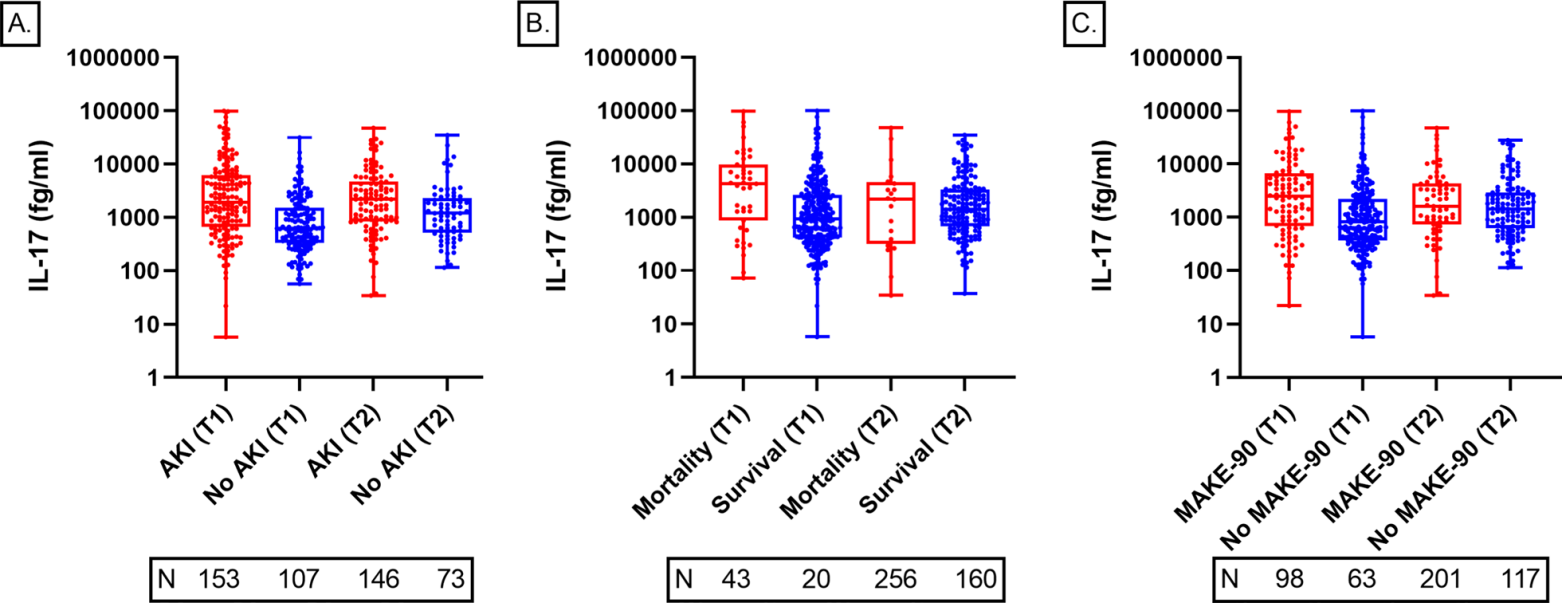
Serum IL-17A levels stratified by acute kidney injury status (Panel A), inpatient mortality (Panel B), and major adverse kidney events (Panel C). T1 = 24–48 h of AKI diagnosis or ICU admission (no AKI); T2 = 5–7 days after initial sample. *N* = number of patients at each represented category. *p < 0.05

**Fig. 2:**
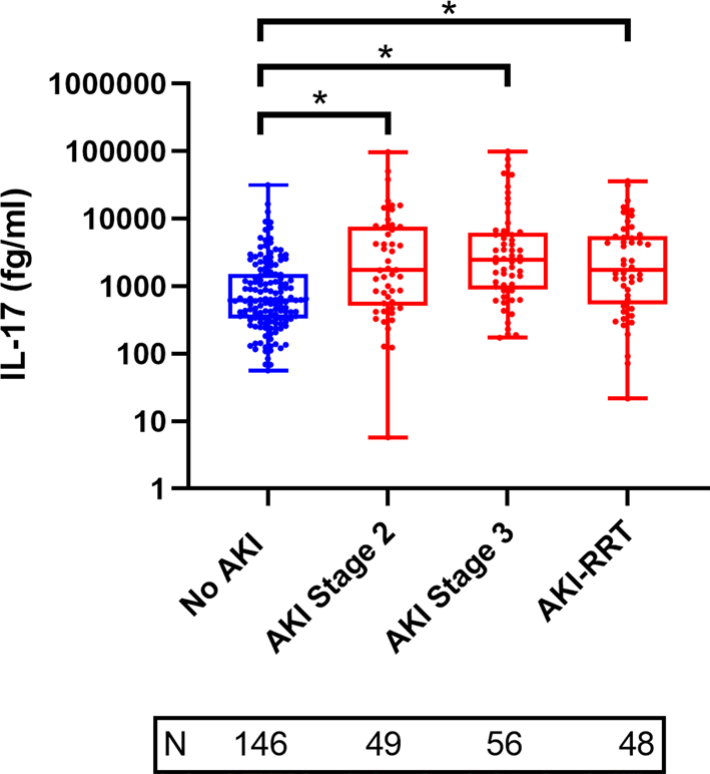
Comparison of IL-17A levels at first timepoint in patients with and without AKI. *N* = number of patients at each represented category. *p < 0.05

Patients with AKI had more prevalent diabetes, liver disease, and anemia when compared to patients without AKI. The Charlson comorbidity index and acuity of illness scores were significantly higher in patients with AKI versus those without AKI (Additional file 1: Table S1). The median (25th–75th percentiles) time from ICU admission to first sample collection was 2 (1–3) days in all patients, 1 (1–2) and 3 (1–4) days for AKI and non-AKI patients, respectively. Patients who suffered AKI had significantly higher levels of IL-17A than those who did not have AKI at both timepoints: T1 1918.6 fg/ml (692.0–5860.9) versus 623.1 fg/ml (331.7–1503.4), *p* < 0.001; T2 2167.7 fg/ml (839.9–4618.9) versus 1193.5 fg/ml (523.8–2198.7), *p* = 0.006. Panel A in Fig. 1 displays the relationship between IL-17A levels in AKI and non-AKI patients at both timepoints. Figure 2 represents that IL-17A levels in patients with AKI stage 2, AKI stage 3 without KRT and those requiring KRT were significantly elevated when these individual AKI severity categories were compared to IL-17A levels in patients without AKI.

### Hospital mortality and MAKE

A total of 43 patients died in the hospital and 3 patients died after discharge within 90 days (mortality rate of 15.4%). Among 253 survivors, there were 30 patients that were determined to be dependent on KRT (26 patients based on EHR data up to hospital discharge and 4 patients based on EHR documentation after discharge). Among 223 patients that were alive and not dependent on KRT up to 90 days post-discharge, 125 (56%) patients had a post-discharge outpatient SCr used for determination of eGFR drop from baseline at a median of 89 (67–103) days from hospital discharge. In the remaining 98 (44%) patients, eGFR drop from baseline was determined using the last SCr during hospitalization.

According to the first timepoint of IL-17A levels, patients that died had significantly higher levels of IL-17A when compared to those that survived: 3058.3 fg/ml (1949.3–4798.7) versus 1080.3 fg/ml (898.1–1299.3), *p* < 0.001 (Fig. 1, Panel B). Patients in the highest IL-17A tertile had 3-times higher mortality rate than those in the lower tertiles: 26% versus 8% versus 9.1%, respectively, *p* < 0.001 (Table 2). Similarly, when evaluating the first timepoint of IL-17A levels, patients that developed MAKE had significantly higher levels of IL-17A when compared to those that did not: 2270.8 fg/ml (1689.4–3052.8) versus 939.5 fg/ml (764.2–1155.1), *p* < 0.001 (Fig. 1, Panel C). The occurrence of MAKE was 2 times higher in patients in the highest IL-17A tertile versus those in the lower tertiles: 53.0% versus 24.0% versus 21.2%, respectively, *p* < 0.001. Among individual components of MAKE, there was a trend toward worse outcomes in patients in the highest tertile of IL-17A versus those in the lower tertiles, although it was statistically significant only for the mortality component (Table 2).

**Table 2:** Study outcomes according to serum IL-17A tertiles

	Tertile 1	Tertile 2	Tertile 3	*p* value
Range, fg/ml	≤ 571.7	586.9–2295.6	≥ 2313.9	
No. of patients	99	100	100	
Primary outcomes				
Hospital mortality, *n* (%)	9 (9.1)	8 (8.0)	26 (26.0)	< 0.001^a,b^
MAKE*, *n* (%)	21 (21.2)	24 (24.0)	53 (53.0)	< 0.001^a,b^
Mortality	9 (9.1)	9 (9.0)	28 (28.0)	< 0.001^a,b^
KRT dependence	7 (7.1)	9 (9.0)	14 (14.0)	0.244
eGFR drop of ≥ 30% from baseline	5 (5.1)	6 (6.0)	11 (11.0)	0.049
Secondary outcomes				
Days in the hospital, median [IQR]	8.5 [5.0–14.8]	11.0 [6.0–20.0]	13.0 [7.0–22.0]	0.010^a^
Days in the ICU, median [IQR]	4.0 [2.0–9.0]	5.0 [2.0–9.8]	8.0 [3.3–15.8]	0.004^a,b^
Days on mechanical ventilation, median [IQR]	0.0 [0.0–2.0]	1.0 [0.0–4.0]	2.0 [0.0–7.8]	0.002^a,b^

*eGFR* estimated glomerular filtration rate, *ICU* intensive care unit, *IQR* interquartile range, *MAKE* major adverse kidney event at 90 days post-discharge, *KRT* kidney replacement therapy

*Frequency of individual parameters for MAKE were evaluated in hierarchical order. First, mortality. Second, KRT dependence in survivors. Third, drop in eGFR in survivors not dependent on KRT

^a^Denotes *p* < 0.05 for comparisons between highest and lowest tertiles

^b^Denotes *p* < 0.05 for comparisons between highest and middle tertiles

In incrementally adjusted multivariable models, every onefold higher serum IL-17A at timepoint 1 was independently associated with an increased risk of hospital mortality (adjusted OR 1.35, 95% CI: 1.06–1.73) and MAKE (adjusted OR 1.26, 95% CI: 1.02–1.55). In the same fully adjusted multivariable models using serum IL-17A tertiles, the highest tertile of IL-17A in reference to the lowest tertile was also independently associated with an increased risk of MAKE (adjusted OR 3.03, 95% CI: 1.34–6.87) but not hospital mortality despite a clear trend (adjusted OR 2.41, 95% CI: 0.92–6.34). There was no significant interaction between IL-17A levels (timepoint 1) and AKI status (AKI vs. non-AKI) for both primary study outcomes (hospital mortality and MAKE), suggesting no effect modification of the reported associations by AKI status (Table 3). When evaluating the value of serum IL-17A at timepoint 2, multivariable mixed linear models of the two timepoints serum IL-17A levels for the primary outcomes revealed a significant interaction between the event occurrence and the timepoint of IL-17A measurements: *p* = 0.047 for hospital mortality and *p* = 0.006 for MAKE. Serum IL-17A levels were distinctively elevated at the first timepoint and remained elevated at the second timepoint in those that died or developed MAKE (Additional file 1: Table S2).

**Table 3:** Multivariable logistic regression of serum IL-17A as the independent variable and (1) hospital mortality and (2) MAKE as the dependent variables

Outcomes	Tertile 1	Tertile 2	Tertile 3	Per onefold higher	Interaction *p* value^#^ IL-17A * AKI status
	aOR (95% CI)	aOR (95% CI)	aOR (95% CI)	aOR (95% CI)	
IL-17A range, fg/ml	≤ 571.7	586.9–2295.6	≥ 2313.9		
No. of patients	99	100	100		
Hospital mortality					0.998
No. of death events	9	8	26		
Model 1	1.00 (ref)	0.86 (0.30–2.45)	3.48 (1.47–8.25)	1.55 (1.24–1.95)	
Model 2	1.00 (ref)	0.82 (0.27–2.48)	2.80 (1.09–7.20)	1.43 (1.12–1.82)	
Model 3	1.00 (ref)	0.75 (0.24–2.34)	2.41 (0.92–6.34)	1.35 (1.06–1.73)	
MAKE					0.926
No. of MAKE events	21	24	53		
Model 1	1.00 (ref)	1.26 (0.63–2.55)	4.31 (2.24–8.32)	1.47 (1.23–1.76)	
Model 2	1.00 (ref)	1.21 (0.58–2.52)	3.51 (1.72–7.14)	1.35 (1.19–1.63)	
Model 3	1.00 (ref)	1.00 (0.41–2.42)	3.03 (1.34–6.87)	1.26 (1.02–1.55)	

MAKE consisted of the composite of death, dependence on renal replacement therapy and eGFR decline ≥ 30% from baseline

Model 1 included age, gender, race, Charlson comorbidity index, and baseline eGFR

Model 2 included variables of Model 1 + non-renal APACHE II and study site

Model 3 included variables of Model 2 + serum creatinine at the time of sample collection (t1)

*aOR* adjusted odds ratio, *MAKE* major adverse kidney events at 90 days post-discharge

^#^Interaction *p* value denotes the statistical interaction between IL-17A and AKI status (AKI vs. non-AKI) for the primary study outcomes

### Secondary outcomes

Patients in the highest tertile of IL-17A had more prolonged ICU stay than those in the lower tertiles: median of 8 [3.3–15.8] versus 5 [2–9.8] versus 4 [2–9] days, *p* = 0.004. In addition, patients in the highest tertile of IL-17A required more days of mechanical ventilation when compared to lower tertiles: median of 2 [0–7.8] versus 1 [0–4] versus 0 [0–2] days, *p* = 0.002 (Table 2). In fully adjusted models, every onefold increase in IL-17A levels independently associated with longer stay in the ICU (*β* 0.07 95% CI: 0.01–0.14, *p* = 0.045), more days of mechanical ventilation (*β* 0.19 95% CI: 0.05–0.32, *p* = 0.008) and overall more days in the hospital (*β* 0.08 95% CI: 0.03–0.14, *p* = 0.005) (Table 4). In a restricted analysis of survivors only, every onefold increase in IL-17A levels also independently associated with longer stay in the ICU and hospital and more prolonged mechanical ventilation (Additional file 1: Table S3).

**Table 4:** Multivariable linear regressions of serum IL-17 as the independent variable and secondary outcomes as dependent variables

Biomarker	Outcome	*β* (95% CI)	*p* value	Interaction *p* value^#^ IL-17A * AKI status
Serum IL-17, per onefold increase	Days in the hospital	0.08 (0.03–0.14)	0.005	0.006
	Days in the ICU	0.07 (0.01–0.14)	0.045	0.108
	Days on mechanical ventilation	0.19 (0.05–0.32)	0.008	0.130

All models were adjusted for age, gender, race, Charlson comorbidity index, baseline eGFR, non-renal APACHE II, study site, and serum creatinine at the time of sample collection (t1)

*ICU* intensive care unit

^#^Interaction *p* value denotes the statistical interaction between IL-17A and AKI status (AKI vs. non-AKI) for the secondary study outcomes

### Sensitivity analysis

Using the 50% eGFR reduction threshold for MAKE, the highest IL-17A tertile (vs. lowest tertile) and every onefold increase in IL-17A levels remained significantly associated with MAKE (Additional file 1: Table S4). These results were concordant with the primary analysis.

## Discussion

The main findings of this study are that (1) serum IL-17A levels measured at the time of AKI diagnosis or ICU admission are differentially elevated in critically ill patients with AKI when compared to those without AKI and that (2) serum IL-17A levels are independently associated with hospital mortality and MAKE, as well as longer requirement of ICU care and mechanical ventilation. This is a large study in humans evaluating this novel biomarker of inflammation in AKI and critical illness.

IL-17A is the primary cytokine secreted from T helper 17 cells (TH17) and plays a key role in host defense, immune modulation, and tissue repair. In this context, IL-17A has been associated with pathology of a variety of diseases. For example, in mice with lipopolysaccharide-induced acute respiratory distress syndrome (ARDS), levels of IL-17A were elevated in plasma, lung tissue lysate, and bronchoalveolar lavage fluid [26]. In the last several years, there has been an increasing number of reports indicating enhanced TH17/IL-17A activation in human kidney disease, while several studies in animal models point to an important role for TH17 cells in kidney disease progression [14]. Several groups have demonstrated that TH17 cells are a major infiltrating lymphocyte in kidneys post-AKI in rodent models [27, 28]. CD4^+^ T cell–deficient mice are protected from cisplatin-induced AKI, whereas adoptive transfer with CD4^+^ T cells restores injury [29]. Our group demonstrated that CD4+ T cells expressing the calcium channel Orai1 exclusively express IL-17A, while CD4+ T cells lacking this calcium channel do not produce IL-17A [29]. Utilizing Orai1 inhibitors to suppress IL-17A expression and promote AKI recovery could represent a new area of AKI therapeutics that needs further investigation.

In our study, serum IL-17A levels were elevated in patients with AKI relative to acutely ill patients without AKI in the ICU, and these levels were also independently associated with hospital mortality and MAKE. Our results are consistent with our previous report demonstrating a fourfold increase in circulating TH17 cells and a tenfold increase in Orai1+ cells in critically ill patients with AKI compared to those without AKI [13] and suggest that kidney injury could potentially activate TH17 cell differentiation. These observations are also supported by Maravista et al., who demonstrated that TH17 cell activation was significantly greater in non-survivors versus survivors of septic shock and AKI [30]. Other studies have reported that TH17 cells or circulating IL-17A are elevated in patients with acute transplant rejection [14] and that persistent elevation in TH17 cells was found in patients with chronic allograft nephropathy [31]. Collectively, these data underpin the potential of IL-17A as a biomarker of AKI that associates with adverse outcomes.

Our study has several important strengths. First, an important characteristic of our study design was to utilize ICU patients without AKI as the comparison group, rather than healthy volunteers without comorbidities (e.g., IL-17A levels from young adult healthy volunteers are ~ 50% lower compared with non-AKI ICU patients in our study, *data not shown*). Second, we had exclusive representation of severe AKI (KDIGO stage 2 or above) in the study. This strategy precludes inclusion of cases of pre-renal azotemia (KDIGO stage 1) which could bias the interpretation of results. Third, the prospective study design allows for timely interrogation of IL-17A and its association with both inpatient and post-discharge outcomes, with emphasis in mortality and MAKE which are important epidemiological outcomes in AKI and critical care. Fourth, the highly sensitive analysis of IL-17A in this study may represent an important technical advantage. We used S-plex plate technology from Mesoscale Discovery (MSD), with high sensitivity to detect serum IL-17A (> 13 fg/ml) 10- to 1000-fold over other assay methods [32, 33]. In contrast, other platforms like U-Plex (MSD) yield undetectable levels of IL-17A in many samples, thus some interrogations of IL-17A and its role in the pathogenesis of many diseases may lack sensitivity, and this potential shortcoming should be considered in future investigations.

Our study also has limitations. First, our sample size restricts the analysis of subgroups of interest according to AKI etiology or cause of ICU admission. Second, we did not include patients with AKI stage 1 and therefore the results may not be generalizable for patients with mild AKI severity. Third, the critically ill population studied has a high mortality rate, and therefore, the MAKE outcome is mostly driven by mortality events, which is a competing risk for the assessment of kidney recovery. Nonetheless, trends of kidney outcomes in survivors according to IL-17A levels were concordant with the results of the composite outcome. Fourth, despite we developed comprehensive multivariable models to test our hypotheses, residual confounding is still possible. Finally, we did not measure other inflammatory biomarkers implicated in TH17 differentiation such as IL-6 and TGF_ß_ or novel biomarkers of kidney injury to further sub-phenotype AKI events [14].

## Conclusion

In conclusion, serum IL-17A levels measured at the time of AKI diagnosis or ICU admission are differentially elevated in critically ill patients with AKI compared to those without AKI and higher levels of serum IL-17A are independently associated with higher risk of hospital mortality and MAKE, and higher ICU resource utilization. The findings described in this study underpin the potential of IL-17A as a valuable biomarker for risk-stratification in AKI and support an important role for this cytokine in the pathophysiology of kidney injury. These results justify further larger prospective investigation of IL-17A as a diagnostic tool and therapeutic target to mitigate inflammation and promote recovery in critically ill patients with AKI.

## Supplementary Information

**Additional file 1:** Supplementary tables.

## Data Availability

Data and materials may be made available upon written request to the corresponding author.
