# Supplementary material for: Serum IL-17 levels are higher in critically ill patients with AKI and associated with worse outcomes
Source: Crit Care. 2022 Apr 14;26:107. doi: 10.1186/s13054-022-03976-4 (PMC9008961; doi:10.1186/s13054-022-03976-4)
Supplement: Supplementary file 1 — Additional file 1: Supplementary tables. [file 13054_2022_3976_MOESM1_ESM.docx]

**Supplementary Table 1.** Patient characteristics according to AKI status

| **Characteristic** | **AKI** | **No AKI** | ***p*-value** |
| --- | --- | --- | --- |
| IL-17A Range, fg/ml | 5.7 – 99007.5 | 56.6 – 31397.7 |  |
| No. of patients | 153 | 146 |  |
| Age, years ± SD | 55.9 ± 15.5 | 57.8 ± 14.9 | 0.282 |
| Women, n (%) | 58 (37.9) | 66 (45.2) | 0.200 |
| Race, n (%)  White  Black  Other | 119 (77.8)  21 (13.7)  13 (8.5) | 116 (79.5)  7 (4.8)  23 (15.7) | 0.008 |
| Body mass index, kg/m^2^ | 30.8 [24.5-37.4] | 27.1 [23.0-32.2] | 0.002 |
| Baseline eGFR, ml/min/1.73m^2^, median [IQR] | 87.9 [74.9-100.7] | 91.7 [80.1-101.2] | 0.203 |
| Baseline SCr, mg/dl, median [IQR] | 0.9 [0.7-1.0] | 0.8 [0.7-0.9] | 0.014 |
| Diabetes, n (%) | 50 (32.7) | 27 (18.5) | 0.005 |
| Hypertension, n (%) | 80 (52.3) | 75 (51.4) | 0.874 |
| CHF, n (%) | 39 (25.5) | 34 (23.3) | 0.553 |
| Liver disease, n (%) | 38 (24.8) | 14 (9.6) | 0.001 |
| Anemia^*^, n (%) | 97 (63.4) | 62 (42.5) | <0.001 |
| Cancer, n (%) | 42 (27.5) | 39 (26.7) | 0.914 |
| Charlson comorbidity index, median [IQR] | 4.0 [2.0-6.0] | 3.0 [2.0-4.0] | 0.001 |
| SCr at first timepoint (t1), mg/dl, median [IQR] | 2.6 [1.7-3.6] | 0.8 [0.6-0.9] | <0.001 |
| CFB 72 h, L, median [IQR] | 3.4 [0.1-7.8] | 1.5 [-0.1 to 3.2] | 0.002 |
| Pressor or inotrope, n (%) | 116 (75.8) | 44 (30.1) | <0.001 |
| Mechanical ventilation, n (%) | 103 (67.3) | 52 (35.6) | <0.001 |
| Packed RBC transfusion, n (%) | 84 (54.9) | 31 (21.2) | <0.001 |
| Non- renal APACHE II score, median [IQR] | 20.0 [15.0-24.0] | 12.0 [9.0-17.8] | <0.001 |
| Non-renal SOFA score, median [IQR] | 8.0 [5.0-11.0] | 4.0 [1.0-6.0] | <0.001 |

^*^Anemia was defined as hematocrit at admission less than 39% in males or 36% in females.

*Abbreviations: APACHE II (acute physiologic assessment and chronic health evaluation II score excluding the renal component), (CFB (cumulative fluid balance), CHF (congestive heart failure), eGFR (estimated glomerular filtration rate), ICU (intensive care unit), IQR (interquartile range), RBC (red blood cell), SCr (serum creatinine), SD (standard deviation), SOFA (sequential organ failure assessment score excluding the renal component).*

**Supplementary Table 2**. Multivariable repeated measures mixed model for serum IL-17A and hospital mortality and MAKE

|  | **Time-point** | **Geometric Mean  (95% CI)** | **Mortality vs. Survival Time 1** | **Mortality vs. Survival Time 2** | **Mortality Time 1 vs. Time 2** | **Interaction** |
| --- | --- | --- | --- | --- | --- | --- |
| 1. Mortality | | | | | | |
| No | T1 | 1080.3 (898.1-1299.3) | *p*=0.016 | *p*=0.458 | *p*=0.211 | *p*=0.047 |
| No | T2 | 1350.2 (1132.2-1610.0) |  |  |  |  |
| Yes | T1 | 3058.3 (1949.3-4798.7) |  |  |  |  |
| Yes | T2 | 2262 (1417.7-3611.6) |  |  |  |  |
|  | **Time-point** | **Geometric Mean  (95% CI)** | **MAKE vs. No-MAKE Time 1** | **MAKE vs. No-MAKE Time 2** | **MAKE Time 1 vs. Time 2** | **Interaction** |
| 1. MAKE | | | | | | |
| No | T1 | 939.5 9764.2-1155.1) | *p*=0.003 | *p*=0.020 | *p*=0.225 | *p*=0.006 |
| No | T2 | 1280.4 (1045.9-1567.4) |  |  |  |  |
| Yes | T1 | 2270.8 (1689.4-3052-8) |  |  |  |  |
| Yes | T2 | 1936.4 (1460.6-2567.3) |  |  |  |  |

Model adjusted for age, gender, race, Charlson comorbidity index, and baseline eGFR, non-renal APACHE II, study site, and serum creatinine at the time of sample collection (t1)

*Abbreviations: CI (confidence intervals), MAKE (major adverse kidney events).*

**Supplementary Table 3.** Multivariable linear regressions of serum IL-17 as the independent variable and secondary outcomes as dependent variables in hospital survivors (n=256)

| **Biomarker** | **Outcome** | **β (95%CI)** | ***p*-value** |
| --- | --- | --- | --- |
| Serum IL-17, per one-fold increase | Days in the hospital | 0.12 (0.06-0.19) | <0.001 |
|  | Days in the ICU | 0.10 (0.03-0.18) | 0.007 |
|  | Days on mechanical ventilation | 0.12 (0.01-0.24) | 0.046 |

Models adjusted for age, gender, race, Charlson comorbidity index, and baseline eGFR, non-renal APACHE II, study site, and serum creatinine at the time of sample collection (t1)

*Abbreviations: ICU (intensive care unit)*

**Supplementary Table 4.** Multivariable logistic regression of serum IL-17A as the independent variable and MAKE as the dependent variable. MAKE consisted of the composite of death, dependence on kidney replacement therapy and eGFR decline ≥50% from baseline

|  | **Tertile 1** | **Tertile 2** | **Tertile 3** | **Per One-Fold Higher** |
| --- | --- | --- | --- | --- |
| **Outcomes** | aOR (95% CI) | aOR (95% CI) | aOR (95% CI) | aOR (95% CI) |
| IL-17A range, fg/ml | ≤571.7 | 586.9 - 2295.6 | ≥2313.9 |  |
| No. of patients | 99 | 100 | 100 |  |
| **MAKE**  No. of MAKE events | 16 | 21 | 45 |  |
| Model 1 | 1.00 (ref) | 1.50 (0.70-3.22) | 4.36 (2.15-8.82) | 1.42 (1.19-1.70) |
| Model 2 | 1.00 (ref) | 1.48 (0.67-3.24) | 3.85 (1.83-8.10) | 1.34 (1.11-1.62) |
| Model 3 | 1.00 (ref) | 1.20 (0.46-3.10) | 3.02 (1.28-7.10) | 1.21 (0.98-1.49) |

Model 1 included age, gender, race, Charlson comorbidity index, and baseline eGFR

Model 2 included variables of Model 1 + non-renal APACHE II and study site

Model 3 included variables of Model 2 + serum creatinine at the time of sample collection (t1)

*Abbreviations: aOR (adjusted odds ratio), MAKE (major adverse kidney events at 90 days post-discharge)*
